# Potassium and the Excitability Properties of Normal Human Motor Axons *In Vivo*

**DOI:** 10.1371/journal.pone.0098262

**Published:** 2014-06-03

**Authors:** Delphine Boërio, Hugh Bostock, Romana Spescha, Werner J. Z'Graggen

**Affiliations:** 1 Department of Neurology, Inselspital, Bern University Hospital and University of Bern, Bern, Switzerland; 2 Sobell Department of Motor Neuroscience and Movement Disorders, Institute of Neurology, University College London, London, United Kingdom; 3 Department of Neurosurgery, Inselspital, Bern University Hospital and University of Bern, Bern, Switzerland; Dalhousie University, Canada

## Abstract

Hyperkalemia is an important cause of membrane depolarization in renal failure. A recent theoretical model of axonal excitability explains the effects of potassium on threshold electrotonus, but predicts changes in superexcitability in the opposite direction to those observed. To resolve this contradiction we assessed the relationship between serum potassium and motor axon excitability properties in 38 volunteers with normal potassium levels. Most threshold electrotonus measures were strongly correlated with potassium, and superexcitability decreased at higher potassium levels (*P* = 0.016), contrary to the existing model. Improved modelling of potassium effects was achieved by making the potassium currents obey the constant-field theory, and by making the potassium permeabilities proportional to external potassium, as has been observed *in vitro*. This new model also accounted well for the changes in superexcitability and other excitability measures previously reported in renal failure. These results demonstrate the importance of taking potassium levels into account when assessing axonal membrane dysfunction by excitability testing, and provide evidence that potassium currents are activated by external potassium *in vivo*.

## Introduction

Nerve excitability tests [1], [2] have been increasingly applied in clinical neurophysiology to assess the excitability properties of motor axons, and to infer likely underlying changes in membrane properties (*e.g.* membrane potential and ion channel functions)[3]–[6]. Recent clinical applications include prediction of survival in amyotrophic lateral sclerosis [7] and early warning of chemotherapy-induced neurotoxicity [8], but an early and continuing contribution has been towards the understanding and possible prevention of uraemic neuropathy. The pathophysiological basis of uraemic neuropathy is not well understood, and unidentified neurotoxic factors have been blamed, but nerve excitability studies have provided evidence that peripheral nerves are chronically depolarized in renal failure, due to hyperkalemia which is only temporarily relieved by dialysis [9]–[13]. This has led to the hypothesis that hyperkalemic depolarization may be an underestimated cause of neuropathy in chronic renal failure [14] and to attempts to prevent the development of neuropathy by maintaining normokalemia.

Despite the importance of potassium effects on peripheral nerve and nerve excitability, the biophysical basis of these effects is only partly understood. Since the resting potential depends primarily on the selective permeability of the axolemma to potassium ions, it is expected that hyperkalemia will cause membrane depolarization with a consequent increase in potassium permeability and membrane conductance, and thereby a ‘fanning-in’ of threshold electrotonus. This behaviour is well accounted for by a model of nerve excitability, in which myelinated axons are represented by two linked compartments (node and internode), with different assortments of ion channels following Hodgkin-Huxley equations [3], [15]–[17]. However this model, which predicts quite well the effects of altering membrane potential by applied currents and the effects of reducing different ion currents, does not account for the effects of hyperkalemia on nerve excitability in end-stage kidney disease (ESKD)[13]. Arnold *et al*. found that the excitability abnormalities in ESKD patients were ‘profoundly worse than that expected for normal axons exposed to similarly high potassium concentrations’. Moreover, whereas superexcitability in the ESKD patients falls steeply with increasing potassium, the model predicts a slight increase [13], because of the reduction in the post-spike hyperpolarization by slow potassium currents. Since their modelling suggested that nodal fast potassium conductance was increased in the patients, Arnold *et al*. proposed that the hyperkalaemia may have disrupted the paranodal myelin, thereby exposing juxtaparanodal potassium channels. However, an alternative interpretation is that their results exposed a deficiency in the modelling of the potassium channels.

There has only been one previous study exploring the relationship between superexcitability and serum potassium in normal subjects (n = 12), which found a significant relationship (p = 0.02), again contradicting the model [18]. The present study was undertaken on a larger group of normal subjects, to define more clearly the potassium dependence of superexcitability and other excitability measures, to clarify any difference from the relationship in uraemic patients, and to improve the model as necessary to account for potassium effects. The results confirm that superexcitability decreases as serum potassium increases, and indicate that potassium currents are more sensitive to external potassium than the present model predicts. A new model is proposed in which the potassium currents in human motor axons depend on extracellular potassium as if 1∶1 binding of potassium ions to the outside of the channel were necessary for their function, a suggestion earlier made to account for the effects of altered potassium concentrations on single myelinated axons of the frog [19].

## Methods

### Ethics statement

All procedures were approved by the local ethical committee: Kantonale Ethikkommission, Bern, Switzerland (KEK-Nr. 180/10), and conformed to the Declaration of Helsinki. Experimental procedures were fully explained and all subjects gave their written informed consent to participate.

### Subjects

Forty healthy volunteers were enrolled to participate in this study. There were 23 women and 17 men, aged between 21 and 79 years. None of the subjects suffered from carpal tunnel syndrome or had any history of a neuromuscular disorder or any risk factor for peripheral neuropathy (including diabetes, neurotoxic medication and alcohol abuse). Finally, subjects with abnormal potassium serum or creatinine were not included. Normal ranges were defined as follows: serum potassium [3.5–4.7 mmol/l] and creatinine [women: 45–84 µmol/l; men: 59–104 µmol/l].

### Laboratory examination

Subjects were comfortably rested on a bed in a warm room. A 5 ml blood sample was taken from one arm and the rest of the examination was performed on the other side. Serum levels of potassium and creatinine were measured. Serum creatinine was used as marker of normal renal function [20].

### Peripheral nerve excitability

Excitability properties of the peripheral nerve were assessed by means of Qtrac software (copyright Institute of Neurology, London, UK), as previously reported [2]. Since temperature affects some excitability parameters [21], cutaneous temperature was carefully monitored and maintained above 32°C through the entire session.

Multiple measures of nerve excitability were performed on the median motor nerve at the wrist, using surface electrodes, as previously described [2], [22], [23]. Electrical stimuli were applied via non-polarizable electrodes (Red Dot, 3 M Health Care, Borken, Germany), the cathode being placed on the median nerve at the wrist and the anode being placed about 10 cm proximal, over the muscle. Stimulus waveforms generated by a computer were converted to current with a purpose-built isolated linear bipolar constant current stimulator (DS5, Digitmer Ltd., Welwyn Garden City, UK) (maximum output 50 mA).

Compound muscle action potentials (CMAP) were recorded from abductor pollicis brevis (APB), using adhesive disposable surface electrodes (REF 9013L0203, Alpine BioMed, Skovlunde, Denmark) with the active electrode at the motor point and the reference electrode on the proximal phalanx. The ground electrode (Red Dot surface electrode) was taped on the top of the hand. The signal was amplified (gain: 1000, bandwidth: 1.6–2 kHz) and digitized with a data acquisition unit (National Instruments NI DAQCARD-6062E, National Instruments Europe Corp., Debrecen, Hungary) using a sampling rate of 10 kHz.

Nerve excitability measurements were made using the TRONDNF protocol. Initially the 1 ms stimulus was set manually to a supramaximal level, and then the computer generated a stimulus-response relationship by progressively decreasing the strength of the stimulus in 2% steps. Next, computer feedback was used to track the stimulus that excited a CMAP equal to 40% of maximal amplitude and threshold comparisons were used to evaluate strength-duration curve, threshold electrotonus, current-threshold (I/V) relationship and recovery cycle. For the strength-duration relationship, the threshold current required to generate the target CMAP was tracked as the pulse duration was reduced from 1 ms to 0.2 ms. During threshold electrotonus, excitability was tested at 26 intervals during and after 100 ms polarizing currents set to ±20% and ±40% of the control threshold current. For the I/V relationship, excitability was tested after 200 ms current pulses that were varied from 50% to −100% of control threshold, in 10% steps. Finally, for the recovery cycle, excitability was tested at 18 inter-stimulus intervals (ISI) from 200 ms to 2 ms after a supra-maximal conditioning stimulus.

### Data analysis

The nerve excitability data were analyzed by means of the QtracP program, as previously described [2]. Multiple excitability measure files were generated for each recording. These files contained all the threshold estimates (*e.g.* for 26 time points on the threshold electrotonus), and also a set of derived excitability measurements that was retained for analysis:

i) from the strength-duration relationship: strength-duration time constant and rheobase

ii) from the threshold electrotonus: mean threshold reductions between the specified times after the start of polarization, for the 40% depolarizing current (TEd40[10–20 ms], TEd40[90–100 ms]), the 20% depolarizing current (TEd20[10–20 ms], TEd20[90–100 ms]), the 20% hyperpolarizing current (TEh20[10–20 ms], TEh20[90–100 ms]), and for the 40% hyperpolarizing current (TEh40[10–20 ms], TEh40[90–100 ms]). Also, the maximal threshold reductions were measured for the 40% and 20% depolarizing currents (TEd40[peak], TEd20[peak]).

iii) from the I/V relationship: resting and minimum I/V slope (an analogue of conductance)

iv) from the recovery cycle: refractoriness, superexcitability and late subexcitability.

### Statistical analysis

All data are reported as mean ±SD. Correlations were assessed by the Pearson product moment correlation coefficient *R*. The level of significance was set at *P*<0.05.

### Nerve models

The dependence of nerve excitability properties on extracellular potassium was modelled in 3 different ways:

#### Model 1

The first model was the human motor axon model described in detail by Howells *et al.* [17]. In this model, potassium and leakage channels are modelled as conductances, as in the original Hodgkin-Huxley model of the squid giant axon, and the first model of human nodal membrane currents [24], e.g.
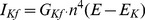
(1)where *I*_Kf_ is the fast potassium current, *G*_Kf_ is the maximum fast potassium conductance (a constant), *n* is the fraction of activated gates, *E* is the membrane potential, and *E*_K_ is the reversal potential for potassium currents, given by the Nernst equation: *E*_K_  =  *RT/F* × ln([K]_o_/[K]_i_). Similarly,
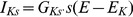
(2)where *I*_Ks_ is the slow potassium current and *s* is the fraction of activated channels, and
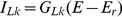
(3)where *I*_Lk_ is the leakage current, *G*_Lk_ the leak conductance, and *E*_r_ is the resting potiential. Equations (1)–(3) are written separately for the nodal and internodal axon membrane. In this model, changes in extracellular potassium only affect potassium currents through their effects on the potassium reversal potential *E*_K_.

#### Model 2

To allow for the fact that potassium ions more readily diffuse from a region of high concentration to one of low concentration than *vice versa*, the potassium currents can alternatively be modelled by the constant field equation, as used by Frankenhaeuser and Huxley to account for the potential dependence of the nodal potassium currents of *Xenopus laevis* [25]. In this formulation, potassium conductances (*G*_K_) are replaced by potassium permeabilities (*P*_K_), and equations (1) and (2) are replaced by equations (4) and (5) respectively:
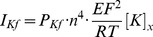
(4)

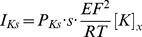
(5)where *F*, *R* and *T* are Avogadro's number, the gas constant and absolute temperature, respectively, and
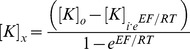
(6)


#### Model 3

Dubois [26] found that for both fast and slow potassium currents at voltage-clamped frog nodes there was a linear relationship between 1/*G*_K_ and 1/[K]_o_ at low values of [K]_o_, consistent with channel opening being dependent on a 1∶1 binding with extracellular K^+^ ions [19]. When [K]_o_ is small, as it is *in vivo*, potassium currents therefore become almost directly proportional to [K]_o_, and this relationship was represented in Model 3 by multiplying [K]_x_ in equations (4) and (5) of Model 2 by the factor [K]_o_/(average [K]_o_).

#### Model fitting procedure

The fitting of the models to the nerve excitability data was performed with the MEMFIT facility in QtracP, which minimizes the ‘discrepancy’ (D), scored as the weighted mean of the error terms: ([x_m_ - x_n_]/s_n_)^2^, where x_m_ is the threshold of the model, x_n_ is the mean, and s_n_ is the standard deviation of the thresholds for the real nerves. To keep the D values consistent in this study, the s_n_ values were always based on the 14 medium K subjects (see below). The weights were the same for all thresholds of the same type (*e.g.* recovery cycle) and were chosen to give total weights to the four different types of threshold measurement: threshold electrotonus, current–threshold relation, recovery cycle, and strength–duration properties in the ratio 2∶1∶1∶0.5. Parameter values for Model 1 were obtained from the recently described model [17] by minimizing the discrepancy between the model and the average of the data from the 38 subjects with an iterative least squares procedure, until alteration of any of the membrane parameters would make the discrepancy worse. For Model 2, starting values of the potassium permeabilities were estimated by making each channel contribution to the resting current the same as in model 1, then the iterative procedure was repeated until the discrepancy was again minimized. Parameter values for Model 3 were the same as for model 2.

## Results

All subjects participated in the study without any adverse effects and none of them requested an early termination of the recording session. However, two subjects had potassium levels outside the normal range (3.1 and 3.4 mmol/l) and were therefore excluded from analysis. Potassium serum levels in the remaining 38 subjects varied from 3.5 to 4.5 mmol/l (average concentration: 4.11±0.25 mmol/l). The subjects were divided into 3 groups on the basis of these potassium levels: Lower K (3.5–3.9, mean 3.82 mmol/l, n = 11); Medium K (4.0–4.2, mean 4.06 mmol/l, n = 14) and Higher K (4.3–4.5, mean 4.39 mmol/l, n = 13). (It should be emphasized that the 3 groups were all within the normal range of 3.5 to 4.7 mmol/l.). Creatinine values were also all within the normal ranges, and varied from 57 to 80 µmol/l (average: 69.4 µmol/l) in women and from 62 to 100 µmol/l (average: 83.2 µmol/l) in men. Average cutaneous temperature at the stimulation site was 32.92±0.73°C. The potassium levels in the subjects were not correlated with age (Pearson *R* = 0.141, *P* = 0.40), temperature (*R* = 0.247, *P* = 0. 14) or sex (*R* = 0.018, *P* = 0.88). However, comparing the younger subjects (14 under 30) with the older ones (24 over 30), although the mean potassium levels were similar in the two age groups (younger 4.09±0.18, older 4.11±0.29 mmol/l, Welch test *P* = 0.79), the variance of the potassium levels was higher in the older group (F test *P* = 0.034).

### Nerve excitability and relation to serum potassium level

**Figure 1:**
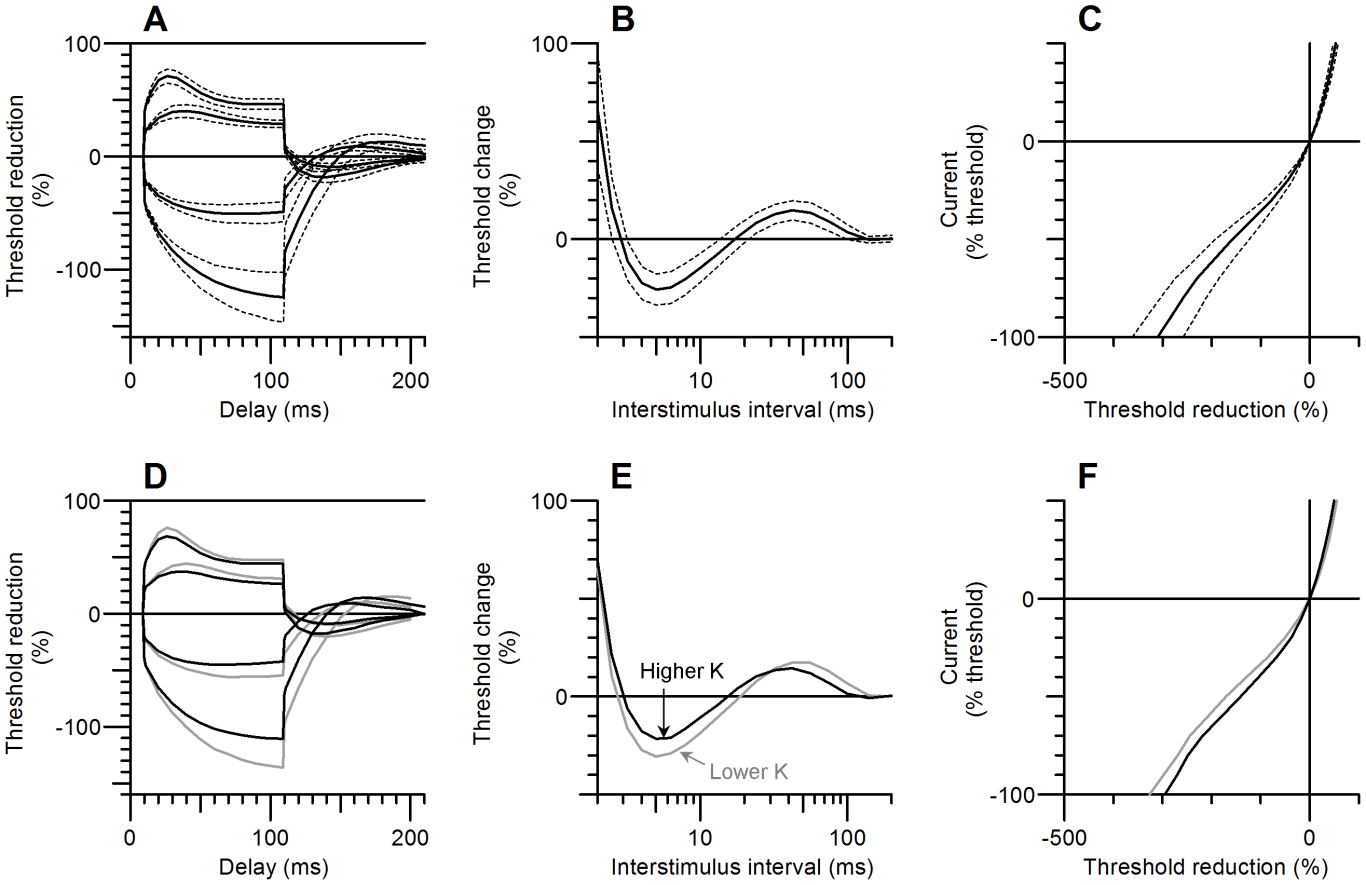
Multiple excitability measurements recorded from normal subjects: motor axons in the median nerve were tested at the wrist and compound muscle action potentials recorded from the abductor pollicis brevis muscle. A–C: Mean +/− SD for all 38 subjects. D–F: Comparisons between means of Lower K (grey) and Higher K (black) groups. A, D: Threshold electrotonus, *i.e.*, threshold changes during and after polarizing currents set to +40 (top), +20, −20 and −40% (bottom) of threshold. B, E: Recovery cycle showing successive phases of refractoriness, superexcitability, and late subexcitability. C,F: Current-threshold (I/V) relationship.

**Figure 2:**
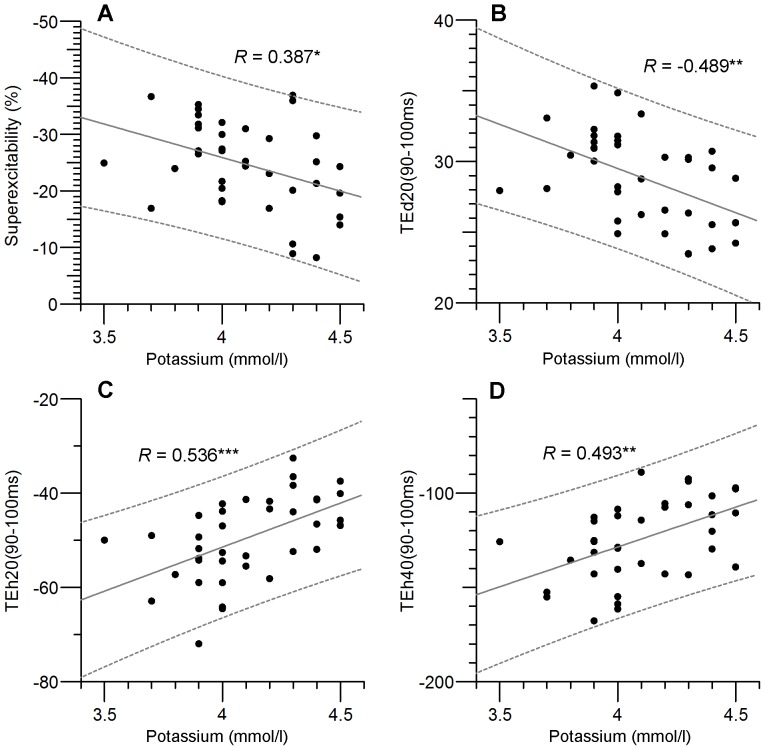
Examples of nerve excitability measures showing significant relationship to serum potassium levels. **A**: Superexcitability, **B**: TEd20(90–100 ms) threshold decrease at end of 20% depolarizing current, **C,D**: TEh20(90–100 ms) and TEh40(90–100 ms) threshold decrease at end of 20% and 40% hyperpolarizing current (NB Negative threshold decrease indicates threshold were increased by hyperpolarization).

Nerve excitability waveforms recorded from the median motor nerve are illustrated in Figure 1 for the 38 subjects. In the top row are plotted the mean waveforms for the 38 subjects ±1 SD. These recordings are very similar to previously published normal median/APB recordings [2]. In the bottom row the mean recordings from the Lower K group are compared with those from the Higher K group. Conventional excitability measurements derived from the waveforms are listed in Table 1, with their correlation to the serum potassium values. As previously reported by Kuwabara and colleagues [18], there was a significant tendency for axons to become less superexcitable at higher potassium levels (R = 0.39, *P* = 0.016). There was a clear tendency for electrotonus to ‘fan in’ at higher potassium levels (*i.e.* TEd values to decrease, TEh values to increase, Table 1 and Figure 2). Over this limited potassium range, there was no significant dependence of rheobase or strength-duration time constant on potassium.

**Table 1:** Mean values of excitability parameters derived from the multiple measures of nerve excitability performed on the median nerve in 38 normal subjects.

	Mean ±SD	*R* v. [K]_o_	*P*
*Strength-duration relationship*			
SDTC (ms)	0.47±0.14	0.187	0.27
Rheobase (mA)	3.84±2.05	0.079	0.65
*Depolarizing threshold electrotonus*			
TEd40[10–20 ms] (%)	69.5±5.8	−0.364	0.024*
TEd40[peak](%)	68.7±5.7	−0.348	0.030*
TEd40[90–100 ms] (%)	46.2±4.5	−0.254	0.12
TEd20[10–20 ms](%)	36.7±4.5	−0.318	0.049*
TEd20[peak] (%)	39.5±3.4	−0.341	0.035*
TEd20[90–100 ms]	28.8±3.2	−0.489	0.0019**
*Hyperpolarizing threshold electrotonus*			
TEh20[10–20 ms] (%)	−38.3±3.6	0.429	0.0070**
TEh20[90–100 ms] (%)	−49.5±8.8	0.536	0.00061***
TEh40[10–20 ms] (%)	−75.8±5.9	0.480	0.0024**
TEh40[90–100 ms] (%)	−124.1±21.6	0.493	0.0018**
*Current-threshold relationship*			
Resting I/V slope	0.597±0.105	0.529	0.00073***
Minimum I/V slope	0.246±0.050	0.097	0.57
*Recovery cycle*			
RRP (ms)	2.88±0.34	0.482	0.0023**
Superexcitability (%)	−24.7±7.7	0.387	0.016*
Late Subexcitability (%)	14.1±4.7	−0.145	0.39

First column shows mean ± standard deviation (SD). Second column shows Pearson product moment correlation coefficient between excitability measure and serum potassium. Third column shows p values (* = P<0.05, ** = *P*<0.01, *** = *P*<0.001). SDTC: strength-duration time constant. TEd20 and TEh20: threshold electrotonus changes due to depolarizing and hyperpolarizing currents respectively, set to 20% of control threshold; TEd40, TEh40 same, but for 40% polarizing currents; expressions in square brackets indicate times after start of 100 ms current, early [10–20 ms], late [90–100 ms] or around peak threshold change [ peak]. I/V: current-threshold. RRP: relative refractory period.

### Comparison with Models 1–3

**Figure 3:**
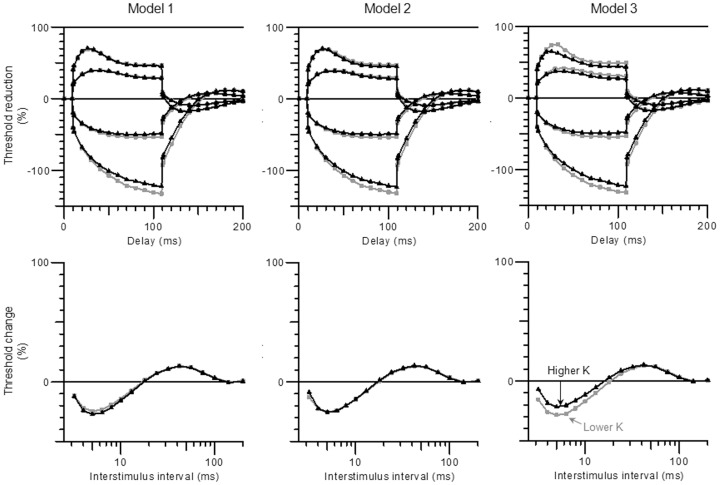
Threshold electrotonus (top row) and recovery cycle (bottom row) waveforms generated by Models 1–3 for values of extracellular potassium corresponding to the Lower K (grey) and Higher K (black) groups.

Figure 3 shows electrotonus and recovery cycle waveforms generated by the three models for potassium concentrations equal to the Lower K (3.82 mmol/l) and Higher K (4.39 mmol/l) groups, which can be compared with the recordings in Figures 1D and 1E. Only Model 3 shows an increase in superexcitability at lower potassium levels and fanning-out of depolarising as well as hyperpolarising threshold electrotonus as seen in the recordings.

**Figure 4:**
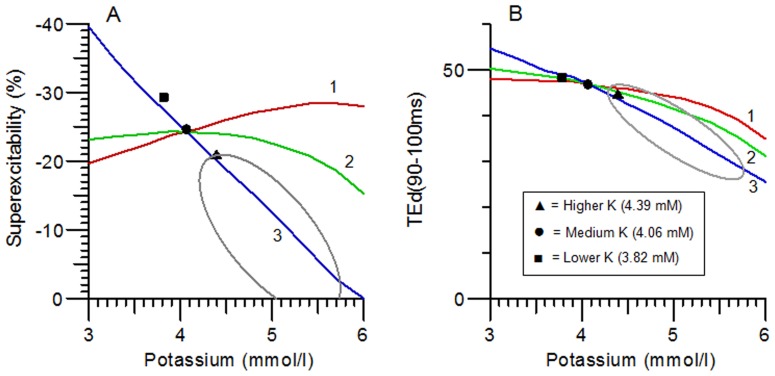
Potassium dependence of 2 nerve excitability measurements predicted by Models 1 (red line), 2 (green line) and 3 (blue line) compared with mean measurements for Higher K (▴), Medium K (•) and Lower K (▪) groups, and ellipse representing 1 SD limits for 9 patients with chronic renal failure (reproduced from Kiernan et al.).^9^ Only Model 3 predicts an appropriate drop in superexcitability with increasing potassium level.

To further explore the potassium dependence of nerve excitability according to the 3 models, and how they predict extrapolation to hyperkalaemic levels, Figure 4 shows 2 excitability measures plotted as a function of potassium concentration, and compares the 3 models with the 3 groups of normal subjects, and also with the previously published data for patients with chronic renal failure, who had varying degrees of hyperkalemia prior to dialysis [9]. In Figure 4A it can be seen that only Model 3 predicts a marked reduction in superexcitability with increasing potassium, and when model 3 is extrapolated to abnormally high potassium levels, it predicts quite accurately the relationship previously found in patients with renal failure prior to dialysis, as indicated by the ellipse. The changes in electrotonus with potassium were too small to distinguish between the models as far as the normal subjects are concerned, but Fig 4B indicates that the changes in depolarizing electrotonus (TEd40[90–100 ms]) in the renal failure patients with hyperkalemia are also best explained by model 3.

Model 3 also provided the best fits taking into account all the excitability measurements (i.e. current-voltage and charge-duration relationships as well as threshold electrotonus and recovery cycle) as judged by the discrepancy scores D (see Methods for definition). The mean data for the Medium K group could be fitted well by either the constant conductance or constant permeability models (D = 0.094 and 0.104 respectively). Without allowance for the differences in potassium, the fits to the Lower K and Higher K group recordings were, not surprisingly, worse (D = 0.412, 0.419 respectively, Table 2). When allowance for the differences in potassium were made according to Model 1, the fits were improved by 14.1% for the Higher K group, but actually made 1.5% worse for the lower K group. Model 2 produced better fits, and Model 3 the best fits, with a reduction in discrepancy of 63.2% for the Higher K group, just by changing the [K^+^]_o_ value from 4.06 to 4.39. Although the discrepancy scores were appreciably higher for the patients with chronic renal failure (who had serum potassium values ranging from 4.3 to 6.1 mmol/l), the discrepancy reductions obtained by the different ways of modelling the effects of the hyperkalemia were similar to those obtained for the Higher K normal subjects (Table 2).

**Table 2:** Comparison between the three models in their ability to account for the effects of changes in serum potassium levels on multiple measures of nerve excitability.

	Lower K^+^	Higher K^+^	High K^+^
	normal subjects (n = 11)	normal subjects (n = 13)	CRF patients * (n = 9)
Medium [K^+^]_o_ (mmol/l)	3.82	4.39	5.02
Discrepancy from Medium K data (n = 14)	0.412	0.419	3.85
Discrepancy from Model 1 (% reduction)	0.418 (−1.5%)	0.360 (14.1%)	3.503 (9.0%)
Discrepancy from Model 2 (% reduction)	0.368 (10.7%)	0.259 (38.2%)	2.545 (33.9%)
Discrepancy from Model 3 (% reduction)	**0.243 (41.0%)**	**0.154 (63.2%)**	**1.218 (68.4%)**

Data from Medium K data was fitted to nerve model, and then adjusted for different potassium levels according to Models 1, 2 and 3. Discrepancies score difference between model and recorded data and discrepancy reductions score improvement over no allowance for potassium. For each data set Model 3 provides lowest discrepancy (figures in bold).

In addition to the Models 1, 2 and 3, we have also explored the consequences of other assumptions about the effects of changes in [K]_o_, for example a constant conductance model with conductances proportional to [K]_o_, and permeability models with only fast potassium channel permeability or slow potassium channel permeability proportional to [K]_o_. These alternative models produced results intermediate between Model 2 and Model 3. Thus Model 3 provided the best simulation of the potassium dependence of both the normal nerves recorded in this study and also the earlier recordings from patients with chronic renal failure.

## Discussion

This study has shown that even within a narrow range of normal serum potassium levels (3.5–4.5 mmol/l), potassium has a significant effect on nerve excitability properties, including superexcitability and the responses to depolarizing and hyperpolarizing currents as measured by threshold electrotonus. In this we have confirmed a previous study by Kuwabara and colleagues [18], using a somewhat different protocol in which 12 normal subjects were each tested on 3 occasions, and in which there was a wider range of potassium levels (3.5–5.0 mmol/l). The principle new finding of this study is that the current model of human nerve excitability cannot account for these relationships. To overcome this deficiency we have presented a new model, in which potassium currents are not only dependent on membrane potential, but also proportional to extracellular potassium concentration. Here we first relate these findings to previous evidence about the potassium dependence of axonal potassium currents, and then consider the implications for future nerve excitability studies.

### Potassium dependence of potassium currents

The dependence on external potassium concentration of potassium currents in frog nodes was studied in detail by Dubois and Bergman [19]. They concluded that the potassium conductance (g_K_) behaved as if proportional to 1∶1 binding of external K^+^ ions to membrane sites, i.e. g_K_  =  G_K_.[K]_o_/(K_app_ + [K]_o_), where G_K_ is the maximum conductance when all sites are occupied, and K_app_ is the apparent dissociation constant. K_app_ depended on membrane potential and external calcium concentration, but was sufficiently high in the physiological region of excitability studies that g_K_ was effectively directly proportional to [K]_o_. The notion of an external binding site was strengthened by the finding that external caesium ions could replace potassium ions in enabling outward potassium currents, so long as their concentration was kept low [19]. That study was re-evaluated by Dubois [26] in the light of his evidence for 3 different types of potassium channel. He concluded that the apparent voltage dependence of K_app_ in the earlier study was attributable to the existence of different potassium channels with different voltage dependence, and that potassium conductance increased with [K]_o_ for both fast and slow potassium channels.

The question of the potassium dependence of potassium currents has received little attention in mammalian myelinated axons. The first nodal voltage clamp studies of rabbit and rat fibers [27], [28] found potassium currents to be almost non-existent, because the fast potassium channels are mainly restricted to the juxta-paranodal region, under the myelin sheath [29], [30]. While later studies of mammalian, including human, nodal ion currents have recognized the importance of slow as well as fast potassium currents [24], [31], [32] the dependence of these currents on external potassium concentrations within the physiological range has never, so far as we are aware, been investigated. Single channel patch clamp studies have found higher unitary channel currents in the high [K]_o_ solutions commonly used than in Ringer solution (*e.g.* 18 ps v. 10 ps for outward ‘I channel’ potassium currents) [33], but possible effects of [K]_o_ on open channel probability have not been described. The present study provides evidence that the potassium channels in human myelinated axons are critically dependent on extracellular potassium, as in the frog.

### Implications for nerve excitability studies

Nerve excitability studies can provide a considerable amount of information about altered nerve membrane properties in disease, but the evidence they provide is indirect and it has sometimes only been by modelling the excitability changes that interpretation has been possible (*e.g.* the effects of sodium channel block by tetrodotoxin) [3]. It is therefore important to ensure that the model can correctly take account of alterations in the nerve milieu, such as potassium concentration, with effects on excitability. In the case of patients with renal failure, the very high correlations found between excitability changes (including superexcitability) and serum potassium levels, provided good evidence of a strong causal connection [9], [10]. Very recently, a causal connection has been proved more decisively by an elegant, two-stage dialysis procedure, in which the serum potassium level was kept constant for the first 3 hours [13]. However, the authors observed that the available model of human motor nerve excitability could not account well for the relationship between serum potassium concentration and excitability properties, especially superexcitability, and suggested that the hyperkalaemia might also be disrupting the myelin sheath. However, our new evidence clearly shows that that model is inadequate to account for the effects of potassium on nerve excitability, even in normal control subjects with potassium levels in the normal range. A better model is required, and the new model presented here provides a simple explanation of how hyperkalemia alone can be responsible for the superexcitability changes in uremia (as illustrated by the ellipse in Fig. 4A) as well as for the dependence of superexcitability on potassium in normal subjects.

The other important lesson of this study is to reinforce the conclusions of Kuwabara *et al.* [17] that excitability studies should be performed when serum potassium levels are stable (*e.g.* before a meal), and where possible a blood sample should be taken at the same time for electrolyte analysis. Model 3 (which is now incorporated in the Qtrac software) provides a means for predicting the likely contribution of serum potassium level to the nerve excitability measurements.
